# Bleeding pattern in the early phase after experimental rotational acceleration induced traumatic brain injury

**DOI:** 10.1007/s00414-025-03457-7

**Published:** 2025-03-18

**Authors:** Daniel Andersson, Kanar Alkass, Julia Anna Mielcarz, Johan Davidsson, Henrik Druid

**Affiliations:** 1 Karolinska Institutet, K7 Onkologi-Patologi, K7 Forskning Druid, Stockholm, 171 77 Sweden; 2 Department of Mechanics and Maritime Sciences, Chalmers University of Technology, Gothenburg, SE - 412 96 Sweden

**Keywords:** Brain, Rat, TBI, DAI, dTAI, Postmortem, Forensic, Hemorrhage

## Abstract

Lethal rotational acceleration induced injury to the brain may leave few detectable intracerebral injuries if the survival time is short. Eighty-two Sprague Dawley rats were utilized in a validated model for standardized rotational acceleration traumatic brain injury to investigate the number and area of subarachnoid and intracerebral hemorrhages. The rats were divided into groups with survival times of 0, 5, 10, 20 and 60 min with equal amounts of experimental and sham operated rats in each group. In addition, a “postmortem” group of rats were euthanizied 5 min before the trauma and samples collected 5 min after the trauma. From all rats, hemispheres were collected, cut and double stained with immunohistochemistry with anti-collagen IV and anti-hemoglobin. Brains from the 20- and 60-minutes groups were stained with immunohistochemistry for amyloid precursor protein beta. The 2 rats with the most and 2 rats with the least intracerebral hemorrhages from all time points were stained for fibrinogen and P-selectin. The group that sustained trauma postmortem and all sham operated rats showed either no bleedings or only a few, minimal, isolated hemorrhages. All other experimental groups showed widespread subarachnoid hemorrhages and few and small intracerebral hemorrhages. The hemorrhages were observed immediately after the rotational brain injury and did not change in number or size during the first hour. Amyloid precursor protein beta staining did not show any convincing axonal accumulation. Fibrinogen and P-selectin showed signs of hemostasis in all antemortem trauma groups. Our conclusion is that hemorrhages from rotatory traumatic brain injury develops immediately upon trauma and do not change during the first hour.

## Introduction

Traumatic brain injury (TBI) occurs when force is applied to the head, producing both structural and functional brain damage, and is a leading cause for morbidity and mortality among the young [1, 2]. Since a large percentage of lethally injured victims die shortly after the trauma [3], there is a need to characterize the early injury pattern.

Rotational acceleration of the head can create multifocal axonal injury by shear forces. The injury is more commonly known as diffuse traumatic axonal injury (dTAI) which can be lethal [4]. In the classification by Adams et al. [5], lethal injuries from dTAI is often grade III with axonal injuries in the subcortical white matter of the cerebral hemispheres, corpus callosum and rostral pons, beyond that dTAI grade III requires a focal lesion in the rostral brain stem. TBI caused by rotational acceleration shows less macroscopic findings than other types of TBI. Certain macroscopic findings may be identified from rotational TBI but may be insufficient in a context of legal medicine, and often only a few small intracerebral hemorrhages are found [6–8].

Primary axotomy, a mechanical event when the axon is disrupted by trauma, is considered rare except in massive axonal injury [7]. Secondary axotomy, a process that takes hours in humans, appears when the axon is partially damaged and represents a neurodegenerative process and may create morphological changes detectable by microscopic examination [9]. Microscopic findings with immunohistochemistry (IHC) for amyloid precursor protein beta (ß-APP) is at present the gold standard for postmortem dTAI-diagnostics but is not entirely specific. Although Hortobagyi et al. [10] reported detectable axonal ß-APP by IHC within one hour, other studies have not observed axonal reactivity for this protein until at least 2–3 h in humans [2, 11, 12].

One of the earliest postmortem signs of a rotatory traumatic injury are hemorrhages, which sometimes may only be microscopically visible. Little is known about the development of the early hemorrhage pattern in TBI caused by rotatory force and it is not clear if the number, sizes and distribution of the hemorrhages can offer information about the injury age and/or detailed information about the force required to produce the injury. In human materials, injury characterization has been reported in postmortem studies, but the levels of rotational force have often remained unknown and the survival times long [6, 13–15].

In the early phase after TBI, acute biomarkers for hemostasis are of interest to evaluate the bleedings. Fibrinogen is cleaved to fibrin by thrombin in the last step of the coagulation cascade and encompasses among other things an important part in the early hemostasis activation contributing to the first aggregation of erythrocytes. Extravasation of fibrinogen also, among other things, contributes to the inflammation later in the TBI injury process [16]. P-selectin is another acute biomarker stored in Weibel-Palade bodies in the endothelial cells and in the alpha granules of thrombocytes. Upon activation and translocation to the cytoplasmatic membrane P-selectin contribute to hemostasis by promoting platelet aggregation and adhesion to injured endothelial cells [17, 18].

In rats the temporal order of events is similar to that in humans in the acute phase but differs in the subchronic and chronic phases [19]. Although one study has reported visible dTAI as early as 5 min after the trauma in rats, the method used is not applicable in postmortem human diagnostics since it requires intrathecal injection of fluorescently tagged dextrans before the trauma [20]. Our model has previously been shown to produce dTAI through sagittal rotation, causing axonal injuries near the brain’s midline structures in the frontal corpus callosum, caudoputamen and the lateral ventricles after survival times of 24 h − 7 days [21], and axonal ß-APP in this model has been detected as early as 2 hours following the trauma [22]. A linear increase of axonal ß-APP expression has been shown to correlate with the rotational acceleration at forces above 1 Mrad/s^2^ [22].

The objectives of this study were to find out how early the hemorrhages are developed, and if their number and sizes will increase with the survival times, up to 60 min. We also wanted to map and compare the distribution of the hemorrhages with the distribution of dTAI that has been studied in this model of rotational acceleration TBI. Since head trauma also may occur among dead bodies, we wanted to find out if the trauma model used would cause bleedings in recently dead rats. Finally, we also decided to investigate if axonal ß-APP could be observed within 60 min after trauma and at which time point signs of hemostasis could be seen.

## Materials and methods

### Animals and the TBI model

A model for rotational TBI which produces sagittal plane angular acceleration as previously described [22], was utilized on 82 Sprague-Dawley rats (weighing 459 ± 78 g, mean ± 2SD ). The exclusion criterion was a survival time not corresponding with the survival time (death > 30 s before or after the allocated survival time given for each rat at the start). In brief, the procedure for both experimental and sham groups was as follows; the skin and periosteum on the upper part of the cranium was divided in the mid-sagittal plane and dissected laterally exposing the bone. An aluminium skullcap was glued to the bone and the head with the skullcap was firmly attached to the movable part, the rotating bar, of the test rig. The resulting center for the sagittal rotation was approximately 1 mm below the base of the skull and 5 mm anterior to the foramen magnum.

In the experimental groups, consisting of 41 rats, an air powered accelerator hit a rubber pad attached to the rotating bar. The force then produced a sagittal acceleration of 1.26 ± 0.73 Mrad/s^2^ (median 1.24 Mrad/s^2^) of the rotating bar. After approximately 30 degrees head extension the bar was decelerated by deforming a block of soft foam. After euthanasia the skullcap was removed with forceps. Except for the absence of trauma, the procedure was identical for the sham group. The animals were anesthetized with a mixture of medetomidin 1 mg/ml, fentanyl 0,05 mg/ml and midazolam 5 mg/ml, 0,25 ml per 100 g body mass before the experiment. The survival times were 0 min (*n* = 7), 5 min (*n* = 7), 10 min (*n* = 7), 20 min (*n* = 7) and 60 min (*n* = 7) ± 30 s after TBI and euthanized by exsanguination. The experiments also included a postmortem group of rats. These were euthanized with pentobarbital and subjected to trauma 5 min postmortem, and their brain samples were collected 5 min after the trauma (*n* = 6). Equal numbers of experimental and sham operated rats were used for all groups. A servo-controlled heating pad was used to maintain the core body temperature at 37.5 ± 0.5^o^C. Heart rate and oxygenation levels were also measured throughout the experiment, both during procedures before trauma and during the survival period. The reason for this was to monitor the animals’ vital parameters and to help with the evaluation of the level of anesthesia.

**Fig. 1:**
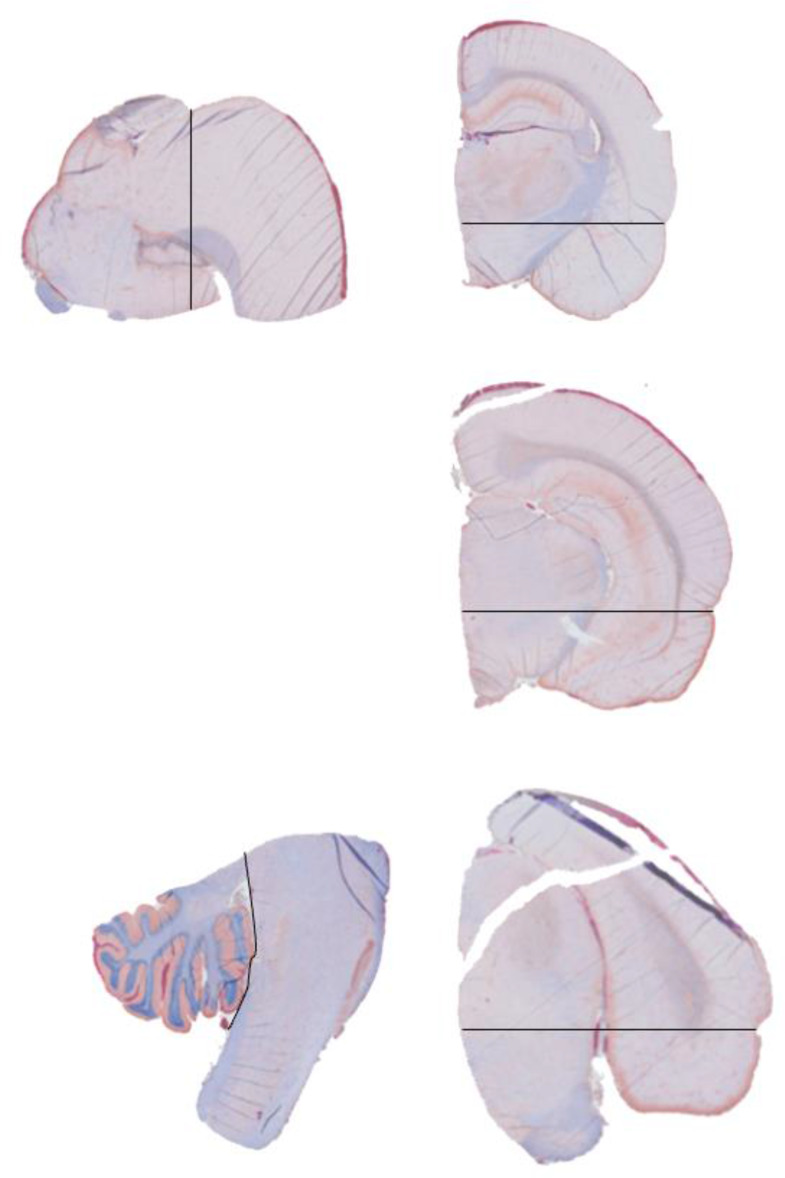
Section from a hemisphere, cut at Bregma 0, -3, -6 and − 10 mm, using a standard cutting frame. The most anterior and posterior blocks were embedded on their medial surface and the coronal blocks on their anterior surface. Black lines indicate the borders of the standardized arbitrary regions, guided by surface landmarks. IHC staining with anti-collagen IV and anti-hemoglobin. Note the wide, thin subarachnoid hemorrhages

Immediately after euthanasia, the skull cap was gently removed with forceps along with the dura mater exposing the dorsal part of the brain. The brain was then removed from the frontal part to rear with a spatula and scissors. The model has previously been shown to produce symmetrical brain damage [21], hence we opted for the following procedure to process the tissue: All brains were divided in the midline using a brain cutting frame. One randomized hemisphere was cut in standardized blocks at Bregma 0, -3, -6 and − 10 producing 5 blocks placed in a histology microcassette that was immediately immersed in 4% phosphate buffered formalin. After the fixation process and paraffin embedding, 4 $\:{\upmu\:}$m thick sections were produced with a rotary microtome. This created five histological standardized brain sections per slide. The most anterior and most posterior blocks were placed in a sagittal position with the midline surface facing down, the other three coronal blocks were placed with the frontal aspect facing down in the cassette (Fig. 1). The reason for the design of the template was the opportunity to identify hemorrhages in the most anterior part of the cerebrum, to allow for the identification of the hemorrhages in the both medial and lateral portions of the cerebrum and to compare amounts and sizes of hemorrhages in the rostral-caudal, and anterior-posterior rhombencephalon. The remaining tissue was stored in paraffin blocks. The contralateral hemispheres were immediately cut as described above, frozen in dry ice and then stored in a -80^o^ C freezer for further studies. Before the trauma, all rats were randomized to either experiment or sham group and survival time.

A sequential double staining of red blood cells and the blood-brain barrier (BBB) basal membrane was performed using anti-hemoglobin and anti-collagen IV. Firstly, the slides were deparaffinated in xylene and rehydrated in a decreasing concentration of ethanol (100% − 70%), rinsed in tap water and then distilled water. Antigen retrieval was carried out by heat-induced epitope retrieval in a decloaking chamber (BioCare Medical) using citrate buffer (0.1 M, pH 6.0). After cooling to room temperature (RT), the sections were washed in water and treated with 0.3% H_2_O_2_ for 10 min to block endogenous peroxidase activity. Blocking serum (4% normal goat serum) (Cat No. S-1000-20, Vector Laboratories) was applied to the slides which were incubated for 30 min at RT. The slides were then washed with PBS with 0.05% tween and incubated at RT for one hour with a polyclonal rabbit anti-collagen IV primary antibody (Cat No. AB6586, Abcam) diluted 1:200. After washing with PBS with 0.05% tween, slides were incubated at RT for an hour with goat anti-rabbit horseradish peroxidase-conjugated (HRP) polyclonal secondary antibodies (Cat No. AP156P, EMD Millipore) diluted 1:250. Thereafter, slides were washed and incubated with ImmPACT^®^ Vector^®^ 3,3’-diaminobenzidine (DAB) substrate kit (Cat No. SK-4100, Vector Laboratories). Next, slides were rinsed with water and incubated with primary rabbit anti-hemoglobin monoclonal antibody (Cat No. AB92492, Abcam) for 1 h at RT. After washing with PBS with 0.05% tween, slides were incubated at RT for an hour with goat anti-rabbit alkaline phosphatase-conjugated (AP) secondary antibodies (Cat No. A0418, EMD Millipore), washed with water and incubated with ImmPACT^®^ Vector^®^ Red aubstrate kit, alkaline phosphatase substrate (Cat No. SK-5105, Vector Laboratories) for visualization. The slides were then counterstained with hematoxylin for 2 min for better visualization of the structures of the tissue, rinsed under running hot water and then distilled water and dehydrated through an increasing concentration of ethanol (70 − 100%). Finally, slides were cleared in xylene and mounted using xylene-based mounting medium in HistoCore SPECTRA (Leica Biosystems). For every staining experiment, a negative control without the addition of primary antibodies was included.

**Fig. 2:**
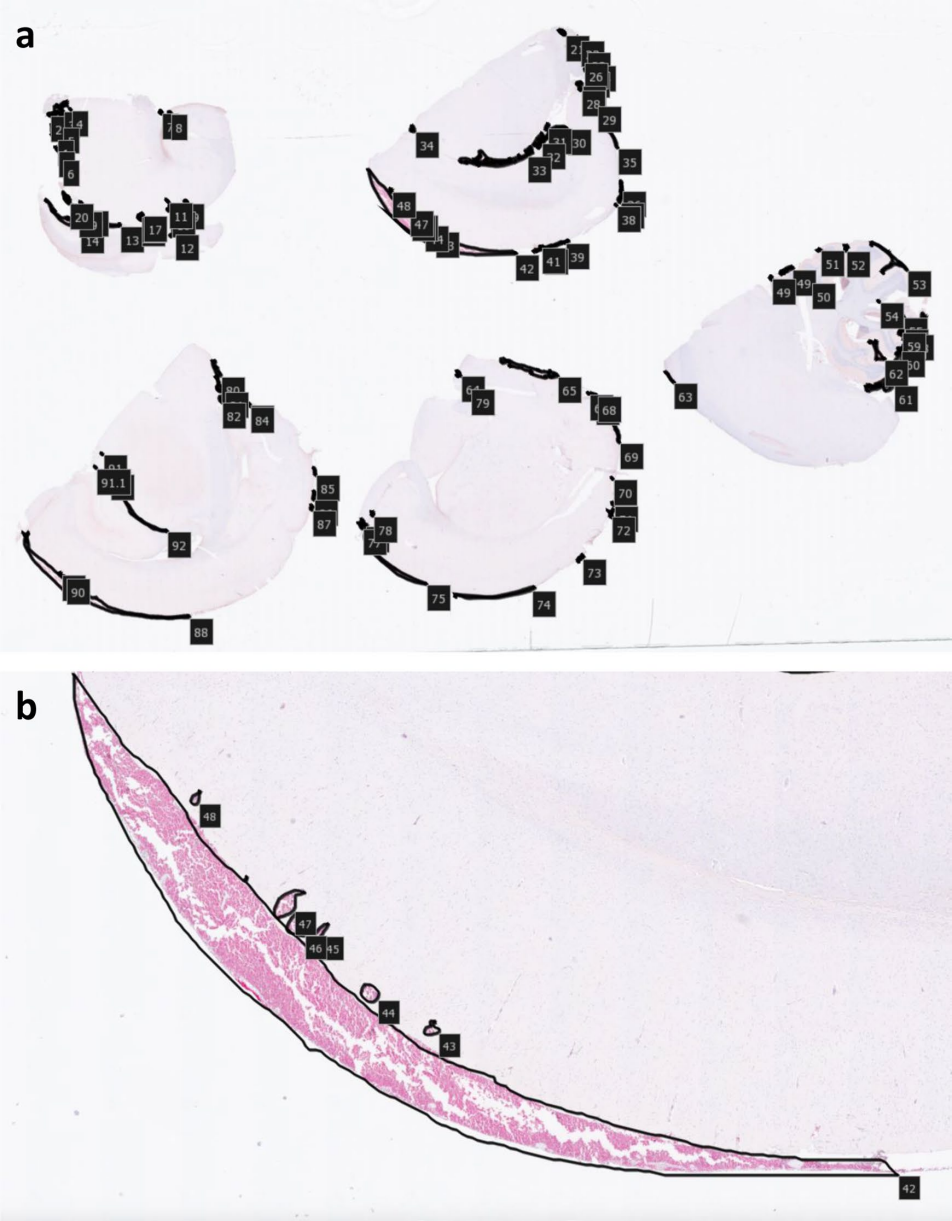
Measurements in the NDP.view2 imaging software showing all areas in an experimental trauma rat with a survival time of 60 min (**a**) with a zoomed area at the dorsal cerebrum at Bregma − 6 (**b**) showing a large subarachnoid and several intracerebral hemorrhages

The double staining with anti-hemoglobin and anti-collagen IV allowed for a reliable identification of bleedings, i.e. red blood cells outside the collagen IV-stained basal layer of the vessel walls. All slides were examined blindly by one observer, and a selection of the hemorrhages was assessed by a second observer. The subarachnoid and intracerebral hemorrhages were manually outlined and their numbers and sizes measured using the NDP.view 2 program (Hamamatsu Photonics K.K., Hamamtsu, Japan). A hemorrhage was defined as presence of erythrocytes outside a blood vessel. If no blood vessel was seen in connection to the erythrocytes an individual assessment was done if the erythrocytes could be artifacts or from a blood vessel not seen in the thin slides. The numbers and areas of the hemorrhages were calculated for each rat and later categorized into groups based on their survival time (Fig. 2a and b).

ß-APP staining was produced in all animals from the 20- and 60-minute groups on separate slides using intelliPATH Biocare Medical with mouse anti-ß-APP (Cat No. MAB348, Millipore) diluted 1:4000. The instrument includes a system with mouse and rabbit secondary antibodies that are conjugated with a polymer to enhance the signal - in our case a HRP-DAB reaction.

Lastly IHC for fibrinogen and P-selectin was performed on separate slides on the 2 rats showing the most and 2 rats with the least intracerebral hemorrhages in each experimental trauma time group, IHC was also performed on 4 of the sham operated rats from the 0 and 20 min groups and the sham operated postmortem group. The deparaffinization, antigen epitope retrieval and the blocking of endogenous peroxidase was performed in the same way as the double staining procedure described above. Blocking serum (2.5% normal horse serum) (Cat No. ZH0913, Vector Laboratories) was applied to the slides which were incubated for 30 min at RT. The slides were then washed with PBS with 0.05% tween and incubated overnight at 4^o^ C with either a polyclonal rabbit anti-fibrinogen primary antibody (Cat No. A0080, Dako) diluted 1:30 000 or a monoclonal mouse anti-P-selectin primary antibody (Cat No. 206002 Biolegend) diluted at 1: 15 000. After washing with PBS with 0.05% tween, slides treated with anti-fibrinogen were treated with horse anti-rabbit HRP conjugated secondary antibodies (Cat No. 30025, Vector Laboratories) and the slides treated with anti-P-selectin were treated with horse anti-mouse HRP conjugated secondary antibodies (Cat No. 30025, Vector Laboratories) and incubated at RT for an hour. Thereafter, slides were washed and incubated with DAB, counterstained with hematoxylin, dehydrated, cleared with xylene and mounted in the same way as for the fibrinogen and collagen IV double-staining. A negative control without the addition of primary antibodies was included for every staining experiment.

The 14 slides with anti-ß-APP staining, the 18 slides with anti-fibrinogen staining and the 18 slides with anti-P-selectin staining were carefully reviewed with bright field microscopy under high magnification for possible axonal ß-APP positivity and evaluation of the hemostasis biomarkers by two independent observers experienced with dTAI-diagnostics and forensic pathology IHC.

### Statistical analysis

A Mann-Whitney U test was performed to evaluate the differences between hemorrhage type, location, numbers and total areas of the hemorrhages in the different survival time groups. The locations were divided into four regions comprising the ventral cerebrum, dorsal cerebrum, rhombencephalon (pons and medulla oblongata) and cerebellum. A horizontal line crossing the dorsal end of the fissura rhinalis was used as the border between the dorsal and ventral cerebrum (see Fig. 1). *P* < 0.05 was considered as statistically significant when comparing groups. Due to multiple tests the *p*-values obtained by Mann-Whitney U test were adjusted with Bonferroni correction. Further, the effect of acceleration on the number and area of both subarachnoid and intracerebral hemorrhages was tested for linear and non-linear correlations (Pearson and Spearman correlation tests). All comparisons of possible differences between groups were first performed applying the Fisher-Pitmans permutations test as this test is robust to non-normal distributions and different variances among groups. This test was performed using R version 4.3.1 applying the *coin* package and the function *oneway test* [23].

## Results

**Fig. 3:**
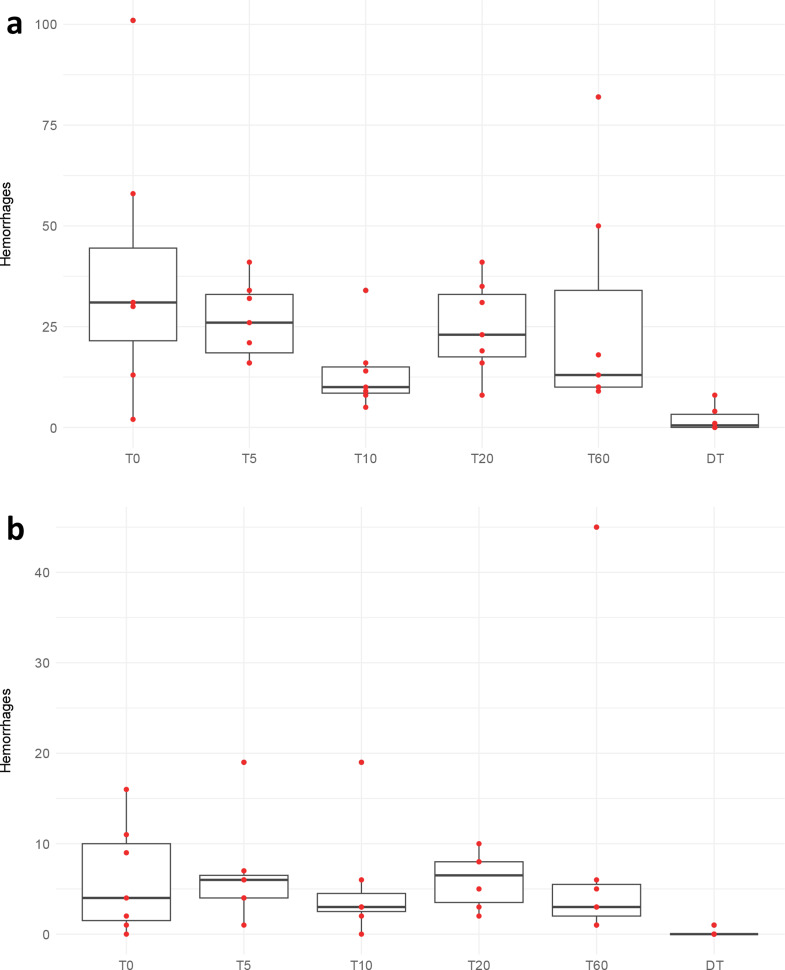
Number of subarachnoid (**a**) and intracerebral (**b**) hemorrhages in the antemortem trauma groups and the postmortem trauma group. Red dots indicate number of hemorrhages for the individual rats; some are overlaying and therefore not distinguishable. T0 = trauma, survival time 0 min, the same for T5, T10, T20 and T60. DT = dead before trauma. Some of the postmortem rats did not show any hemorrhages. T0 = trauma, survival time 0 min, the same for T5, T10, T20 and T60. DT = dead before trauma

**Fig. 4:**
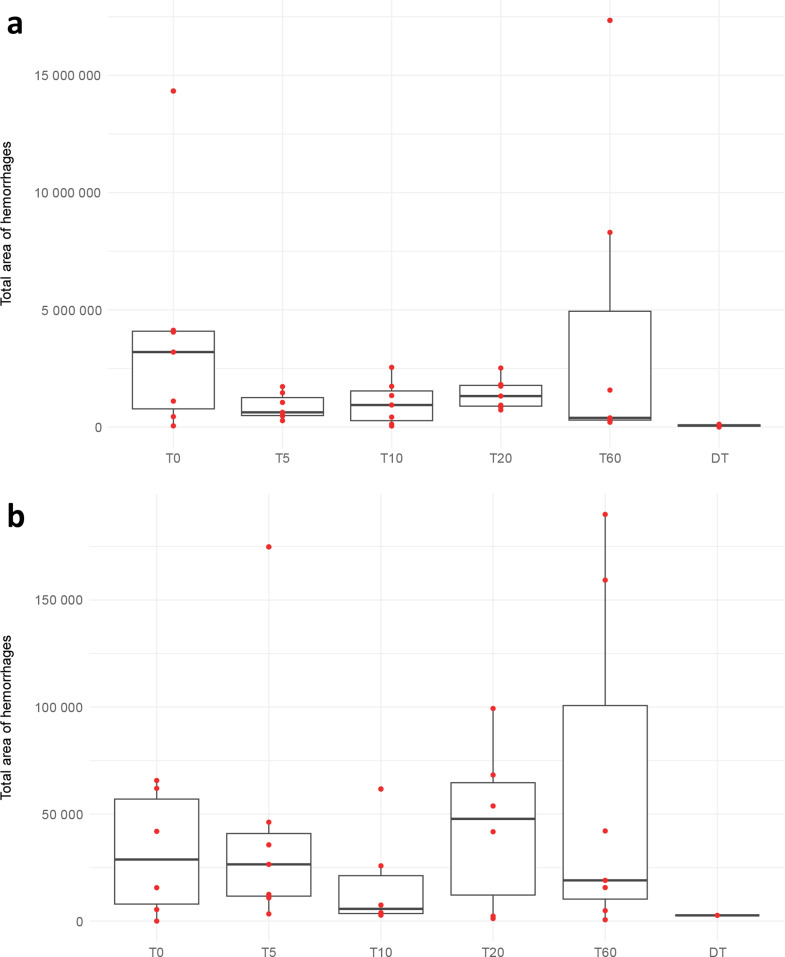
Areas of the subarachnoid (**a**) and intracerebral (**b**) hemorrhages. Red dots indicate the total area of the hemorrhages for the individual rats; some are overlaying and not distinguishable. Some of the postmortem rats did not show any hemorrhages. T0 = trauma, survival time 0 min, the same for T5, T10, T20 and T60. DT = dead before trauma

**Fig. 5:**
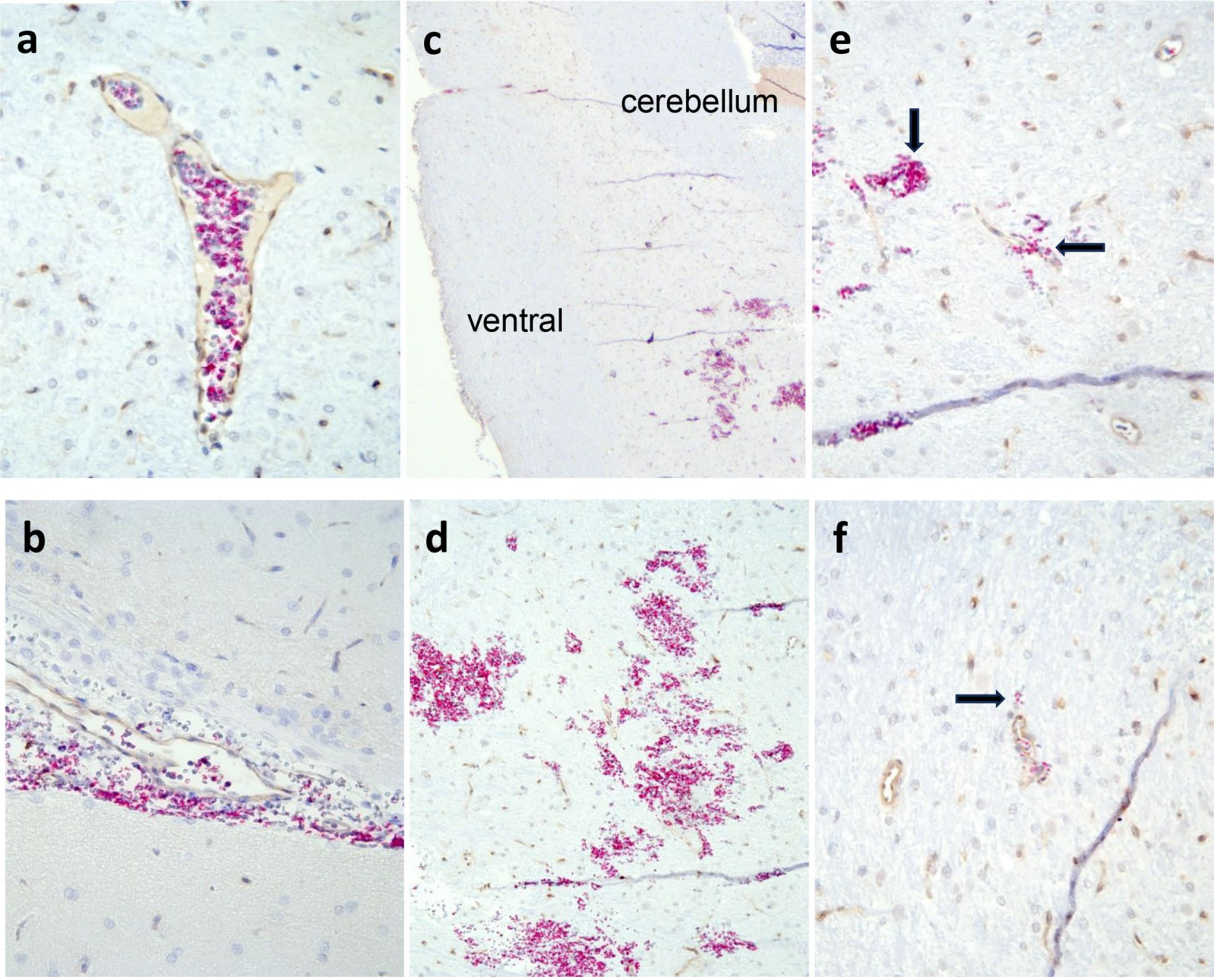
Micrographs of an IHC double-staining with anti-collagen IV and anti-hemoglobin. **a**) intact small vein, **b**) subarachnoid bleeding, **c**) a group of bleedings in medulla oblongata, **d**) a magnification of these hemorrhages, **e**) small hemorrhage between two capillaries (long arrow) and outside a venule (short arrow), **f**) even small hemorrhages were counted (long arrow) by marking the borders and measuring the area with the NDP.view2 imaging software. Original magnification 25x (**c**), all others 400x

The experimental antemortem trauma groups showed numerous and widespread subarachnoid hemorrhages (Figs. 3a and 4a) whereas the intracerebral hemorrhages (Figs. 3b and 4b) were few and small and were always seen close to small blood vessels; capillaries and venules and rarely arterioles (Fig. 5). No statistical differences were seen in the number or area of the bleedings between the different survival times groups. No correlation was found between the rotational acceleration and the number of hemorrhages, and there was no statistical difference in acceleration force between groups. However, three rats died due to the trauma before the experimental endpoint, and they did experience somewhat higher acceleration force than the averages of their designated groups and the average of all groups. Their number and size of hemorrhages were also somewhat larger than the averages, but there were other rats surviving their designated survival times with higher hemorrhage numbers and areas.

The antemortem sham groups and the postmortem experimental and sham groups showed only a few, minimal hemorrhages located at the periphery of the tissue.

Intracerebral hemorrhages in the experimental antemortem trauma groups were most frequently found in pons, cerebellum, corpus callosum, and in the subcortical areas of cerebrum. Significantly more and larger intracranial hemorrhages (*p* < 0.05) were seen in the rhombencephalon and cerebellum compared to the dorsal and ventral cerebrum, when related to the area of the subdivisions (Table 1). Similar to human rotational TBI, the bleedings in pons were predominantly found rostrally and in the posterior part, and the bleedings in corpus callosum were typically found at the border to the parasagittal white matter. Hemorrhages in the medulla oblongata were seen in some rats (Fig. 5c, d).

**Table 1:**
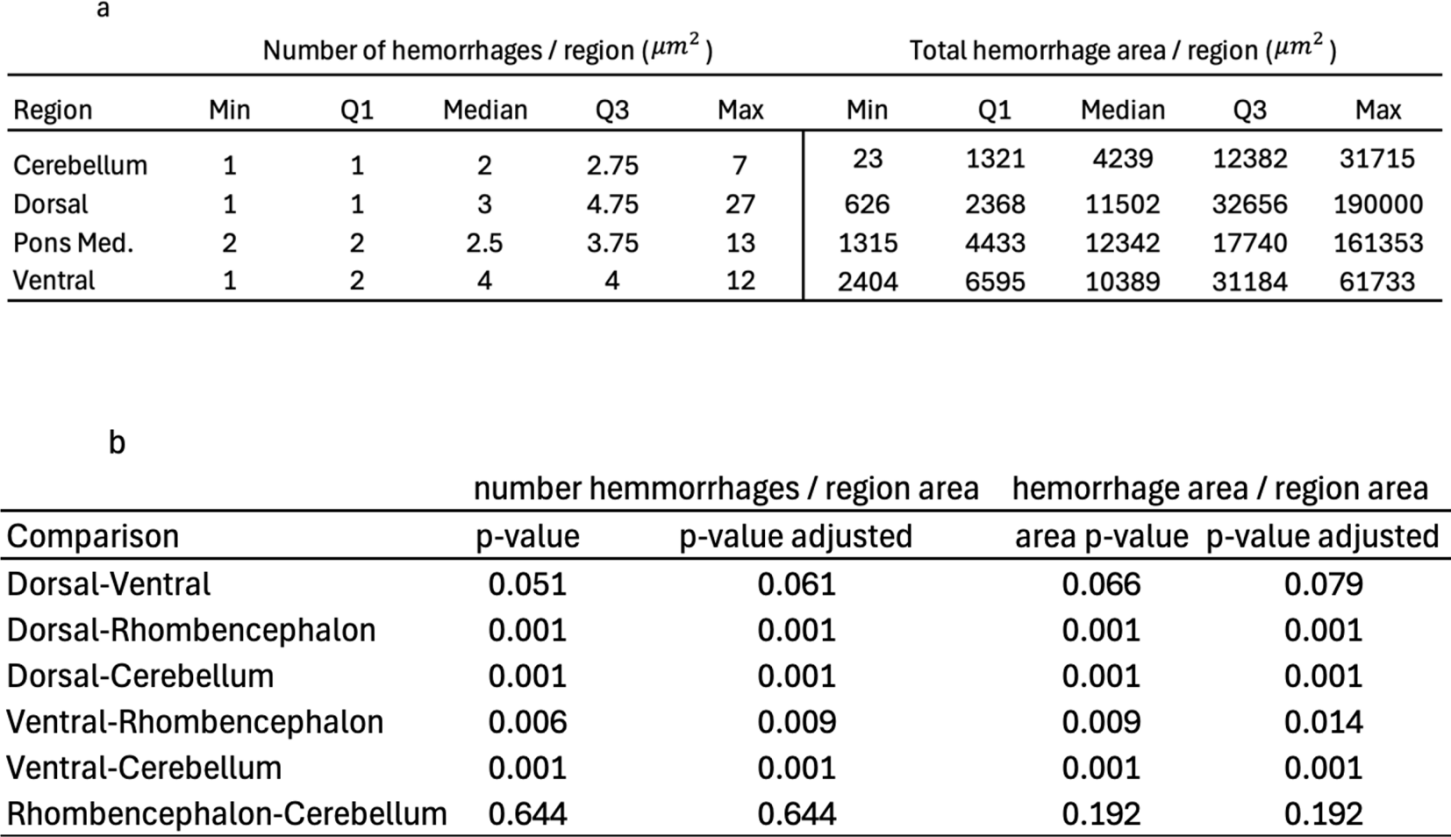
Amount and areas of intracebral hemorrhages of all antemortem experimental trauma groups per region (**a**) and comparisons between regions (**b**)

**Fig. 6:**
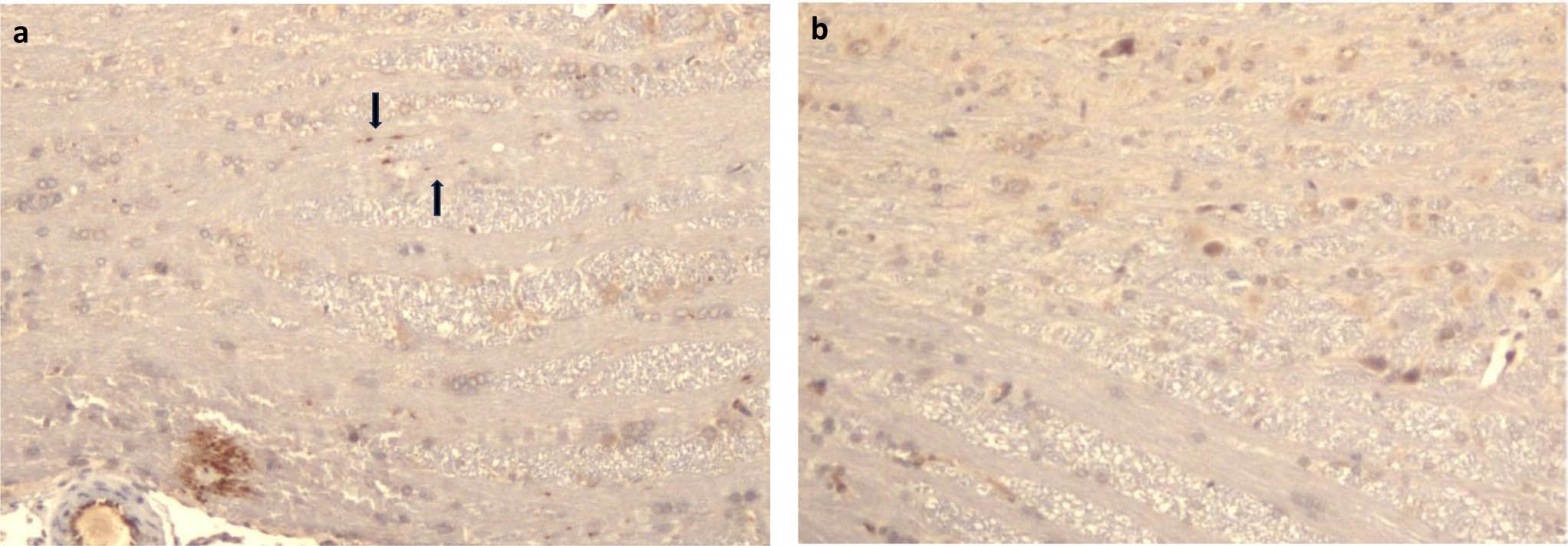
High power micrographs, 400x, from pons of an antemortem 60 min trauma rat. **a**) minute ß-APP positivity in suspected axons (arrows). **b**) same area of a sham 60 min rat. Note normal ß-APP positivity in neuronal cytoplasm

Immunohistochemical examination of anti-ß-APP expression showed consistently a marked positivity in neuronal cell somata. Minute, punctate positivity in one or two putative axons (Fig. 6a) was only observed in one experimental 60 min rat, all other experimental and sham operated rats, or postmortem rats, showed no signs of ß-APP accumulation in the axons (Fig. 6b).

**Fig. 7:**
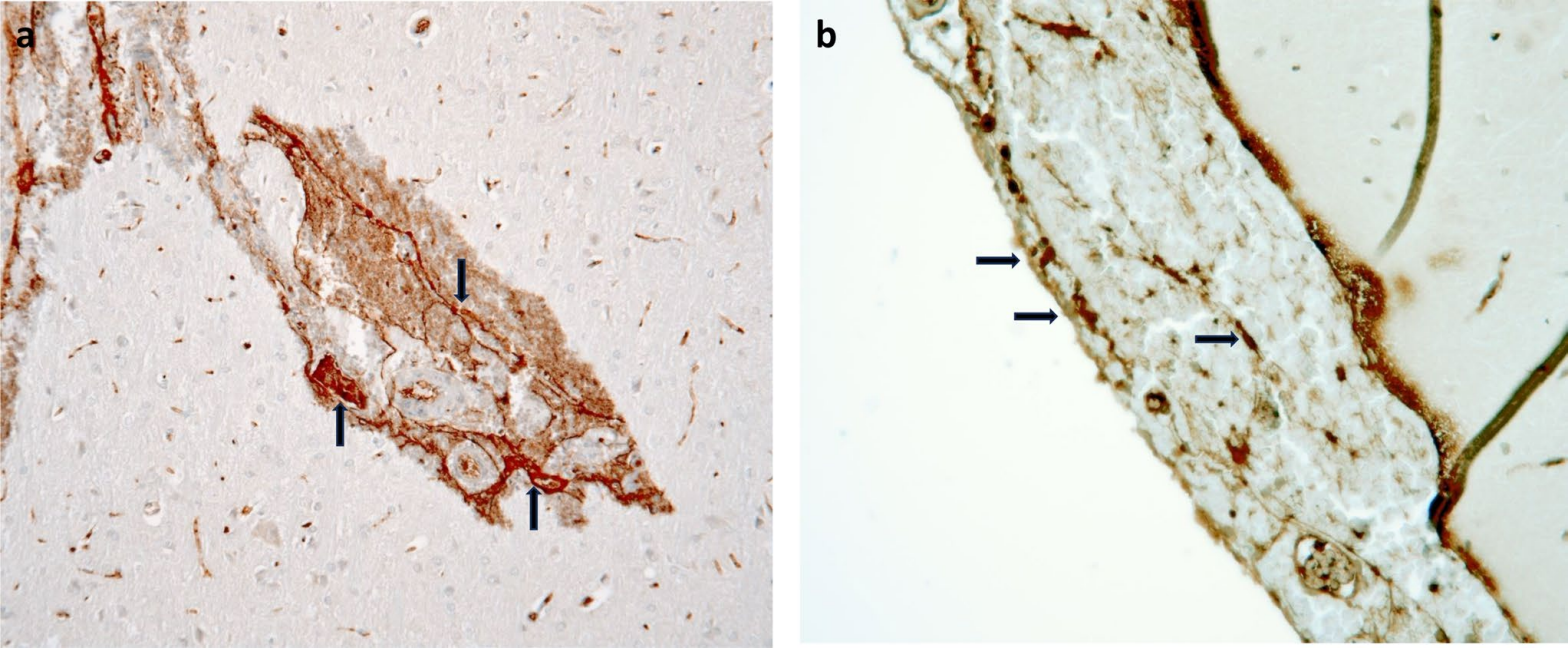
Micrograph of an **a**) intracerebral hemorrhage from a rat with a survival time of 0 min stained for fibrinogen and **b**) subarachnoidal hemorrhage stained for P-selectin. Arrows are showing accumulation of fibrinogen and P-selectin in network formations

Anti-fibrinogen staining showed network formations in the hemorrhages (Fig. 7a) in all survival times in the antemortem trauma groups. No difference in the staining pattern was seen between the groups. Positivity was also seen in the capillaries. The group receiving trauma postmortem and all sham operated groups showed positivity in the capillaries. Only one small subarachnoid hemorrhage was identified in one sham operated rat with a survival time of 0 min on the dorsal part of the brain with fibrinogen network formation. In the hemorrhages and in many of the the small vessels, particularly at the vessel walls, of the antemortem trauma groups aggregates of platelets were seen, often forming networks, suggestive of adhesion to fibrin strands (Fig. 7b). No differences in the staining pattern was perceived within or between the groups by the observers.

## Discussion

The subarachnoid and intracerebral hemorrhages developed within the first minute after the trauma and did not change in numbers or sizes during the first hour between the groups. To our knowledge, there are no previous studies on the development of hemorrhages during the first hour after rotational trauma. Although most hemorrhages were small, particularly the intracerebral hemorrhages, it was somewhat surprising to find that they appear immediately after trauma, given that the rats in all groups were rapidly exsanguinated and then decapitated, procedures that are expected to reduce the difference in intra- and extravascular pressures in the brain. The robustness of the study with a model producing standardized trauma evaluated at several given time points greatly contributed to the assessment.

The small hemorrhages at the periphery of the tissue in the experimental sham groups might represent artifacts produced during the removal of the brain. Such bleedings were also seen in the rats that received trauma. Even though these may have been produced during removal/handling of the brain, they were consistently counted as true hemorrhages in all rats. No reactive changes were seen at the borders of the bleedings, and there were no signs of edema in neuropil. 

In the group that received trauma 5 min postmortem either no or only a few minimal hemorrhages were seen, most of which were superficial and might have been produced during the removal of the brain. The tissue in this group was collected 5 min postmortem which makes hypostasis as a cause for the hemorrhages very unlikely, since much longer time in a nose-down position would be required according to the studies by Xiang et al. [24]. Since this group’s bleeding pattern greatly resembled all the sham operated groups our interpretation is that the trauma was not the cause of the scarce small, isolated superficial hemorrhages observed. Translated to the forensic casework, our results suggest that scattered, minute intracerebral bleedings are not likely to be caused by a postmortem head trauma; hence a body with severe head injuries with very few, or with no bleedings at all should raise suspicion of postmortem trauma.

An explanation as to why the intracerebral hemorrhages did not expand with increasing survival time between the groups may be that further bleeding was counteracted by the resistance by the space-occupying hemorrhage in the neuropil surrounding the small, ruptured vessels. In the subarachnoid space, the tissue is less dense and the explanation for absent bleeding expansion of the subarachnoid bleedings remains unclear. In both cases, an immediate sealing may take place by e.g. contraction, or patching [25] if the opening is small, and somewhat larger defects may rapidly be covered by a primary platelet plugs. This notion is supported by our observation of both platelet aggregates and fibrin networks already in the trauma 0 min group. These observations may be of importance in the routine forensic casework since they suggest that more numerous bleedings, or larger bleedings, found at autopsy of a victim of rotational trauma more likely indicate a higher force rather than a longer survival interval.

We used large and arbitrary regions for the geographical mapping of the intracerebral hemorrhages, since the hemorrhages were too few to make smaller subdivisions meaningful, and we then used the same regions for recording the localization of the subarachnoid hemorrhages. The scarcity of the intracerebral hemorrhages is in agreement with forensic pathology experience and publications on human dTAI [5–7]. Although the intracerebral hemorrhages were grossly seen at locations observed in dTAI in humans [5], the scarcity of the intracerebral hemorrhages did not allow for a detailed comparison between their presence in these regions with published data on dTAI localization. Some subarachnoid and intracerebral hemorrhages were co-localized, but all of them were separated by intact brain tissue, at least as far as it could be judged from the 4 $\:{\upmu\:}$m thick sections studied. Experimental studies of the early hemorrhage patterns are difficult to find for comparison with the results in our study; most studies have used much longer survival times. In a model in rabbits that received severe rotational TBI, extensive subarachnoid hemorrhages and small intracerebral hemorrhages were seen after one hour [26, 27]. Although the hemorrhages in that model were not characterized in detail, the overall pattern was seemingly similar to the numbers and localization in this study.

Although the permutation test of multiple comparisons did not indicate difference between regions, the Mann-Whitney U test with Bonferroni correction showed that there were significantly more hemorrhages and larger hemorrhage areas in the rhombencephalon and cerebellum compared to the dorsal and ventral cerebrum when related to the area of the subdivisions (Table 1). These findings should be interpreted with caution since the area of the structures with more hemorrhages and larger areas had much smaller subdivisions than the cerebral subdivisions. Furthermore, the hemorrhages were in general few and small in all blocks, so the only firm conclusion from these results is that the overall pattern show similarities with the distribution of dTAI grade III in humans.

The cutting of the brain was designed to allow for several different analysis in order to also conform to the 3R animal harm reduction strategy. Hence, the anterior and posterior blocks were embedded on the medial side (Fig. 1) for microtome cutting, which implies that their lateral parts were not included in the hemorrhage assessment. However, since we did include the lateral parts of the three intermediate blocks, we believe that the distributions of bleedings in the lateral parts of the anterior and posterior blocks are similar. Another drawback with the cutting scheme is that the blocks are fairly thick. Hence it is possible that some subarachnoid bleedings longer than 3 mm in the anterior-posterior direction may have been observed in two blocks and hence been counted as two separate bleedings. An alternative cutting of the brain could have been to cut several more, thinner coronal blocks and to consistently examine their anterior aspect, which would allow for examination of more regions. However, we believe that a more extensive microscopic evaluation would not add much to the actually observed overall hemorrhage pattern.

Another question to be addressed is the difference between the human and rat brain and how our findings grossly can translate to human TBI. The rat brain is lissencephalic compared to humans’ gyrencephalic brain. A gyrencephalic brain deforms more easily than a rat brain, and the gyri and sulci contribute to create more uneven stress. Further, the small size of a rat brain compared to a human brain implies that the trauma will be less severe in rats at a given angular acceleration [28]. Compared to a human brain, the level of rotational acceleration and deceleration in our model was predominantly moderate to severe (median 1,24 Mrad/s^2^); Three rats died due to the trauma; these were excluded since they did not survive the full time in their allocated group. We did nevertheless examine their bleeding patterns, which by and large were similar to the other rats subjected to trauma. The rotational acceleration for these three rats was close to the median for all trauma rats.

In addition to the size and anatomical differences, the time for various physiological and biochemical changes to take place differ between rodents and humans. The rate of metabolism, physiological functions and cellular processes are approximately 2.5 to 84 times faster in rodents than humans [19]. Hence, even if the injury pattern and sequence of events may be similar, all changes will occur at a slower rate in humans.

In order to separate erythrocyte aggregates inside vs. outside vessels to identify bleedings, we first tried IHC staining of erythrocyte membranes with the glycophorin (GP) family membrane protein known to have similar properties in humans as in rats [29]. Three different antibodies for glycophorin A (GPA) and one for glycophorin B (GPB) did stain erythrocyte membranes well on human postmortem samples, as previously reported [30–32] but none of them showed appropriate staining on the rat samples despite that the manufacturers indicated cross-reactivity. Hence, we instead used an anti-hemoglobin antibody that worked well together with an anti-collagen IV antibody that visualized the basal membrane of the vessel walls. We did not experience any problems with artifactual erythrocyte aggregates due to microtome cutting, probably because of the exanguination before termination.

Intracerebral bleeding is an obvious sign that the BBB has been damaged. In TBI in humans the BBB disruption is biphasic and the first peak occurs within the first hours [4, 33]. Our results show that the BBB is disrupted immediately after rotational TBI in rats and this is likely to be true in humans after a similar trauma. Given that the intracerebral bleedings are scarce and small, and that the somewhat larger subarachnoid bleedings will not per se leak out molecules specific to the brain, elevated blood or CSF levels of brain injury biomarkers are not likely to be found in the very early phase after a rotational brain trauma. In addition to the damaged BBB, the recently discovered glymphatic system may also contribute to the translocation of the biomarkers outside the central nervous system (CNS) [34], but this system needs further studies to be fully understood. The knowledge of the hemorrhage development can be of value not only from a forensic medicine perspective but also to clinicians and the basic science researchers trying to understand the early mechanisms of TBI. For instance, extravasated blood in the neuropil has been shown to promote inflammation in the brain [35, 36].

The anti-fibrinogen staining showed network like positivity in the hemorrhages in all antemortem trauma groups which shows that the hemostasis starts almost immediately upon the vessel wall injuries. The acute hemostatic mechanism in combination with a pressure from the hemorrhages on the injured, small vessels may explain why the hemorrhages did not increase in size or numbers with increasing survival times. It was not possible for the observers to differentiate P-Selectin positivity in the cytoplasm from the cell membrane. A reason for this may be too high concentration of the primary antibody but also the small size of the endothelial cells and the platelets. Single staining with P-selectin has been shown to be unspecific in diagnosing intravital injuries [18]. However, aggregates of P-selectin positive platelets were seen in the hemorrhages and at the walls of many small vessels in all antemortem trauma groups, but not in the postmortem group. A way to make a more specific assessment of P-selectin in the future could be to use it’s ligand PSGL-1 with the proximity ligation assay. One sham operated rat with a survival time of 0 min showed network like positivity for fibrinogen in a small subarachnoid hemorrhage on the dorsal part of the cerebrum. The model have previously shown to produce injury near the area for surgery which may be an explanation for the observations of occational small hemorrhage at this location [21].

It should be kept in mind that in human studies, the hemorrhages are often, but not always, co-localized at the exact same location as the dTAI [13]. We were unable to visualize any convincing axonal injuries in any of the tissue array samples of the trauma rats, neither with conventional histology nor with IHC with anti- ß-APP, except for a minimal suspected positivity in the pons of one rat (Fig. 6a). Given that biochemical processes occur much more rapidly in rats than in humans, our results suggest that ß-APP positivity in human brain samples most likely indicate a survival interval significantly longer than 1 h.

Our main conclusion of the study is that hemorrhages from rotatory TBI develops immediately upon trauma and do not change during the first hour in rats. Extensive numbers of bleedings and/or large bleedings are more likely to be due to a more severe head trauma than a long survival time, which has implications for the interpretation of brain hemorrhage patterns in unwitnessed deaths.

## Data Availability

The datasets generated during and analyzed during the current study are available from the corresponding author on justifiable request.
